# Correction: Bounds on Transient Instability for Complex Ecosystems

**DOI:** 10.1371/journal.pone.0162430

**Published:** 2016-09-01

**Authors:** Francesco Caravelli, Phillip P. A. Staniczenko

The following information is missing from the funding statement: PPAS was supported by an AXA Research Fellowship, British Ecological Society grant 4785/5824 and the National Socio-Environmental Synthesis Center (SESYNC)—NSF award DBI-1052875.

The ϵ labeling the key for Fig 1 should be replaced with the symbol r. The authors have provided a corrected Figure here.

**Fig 1:**
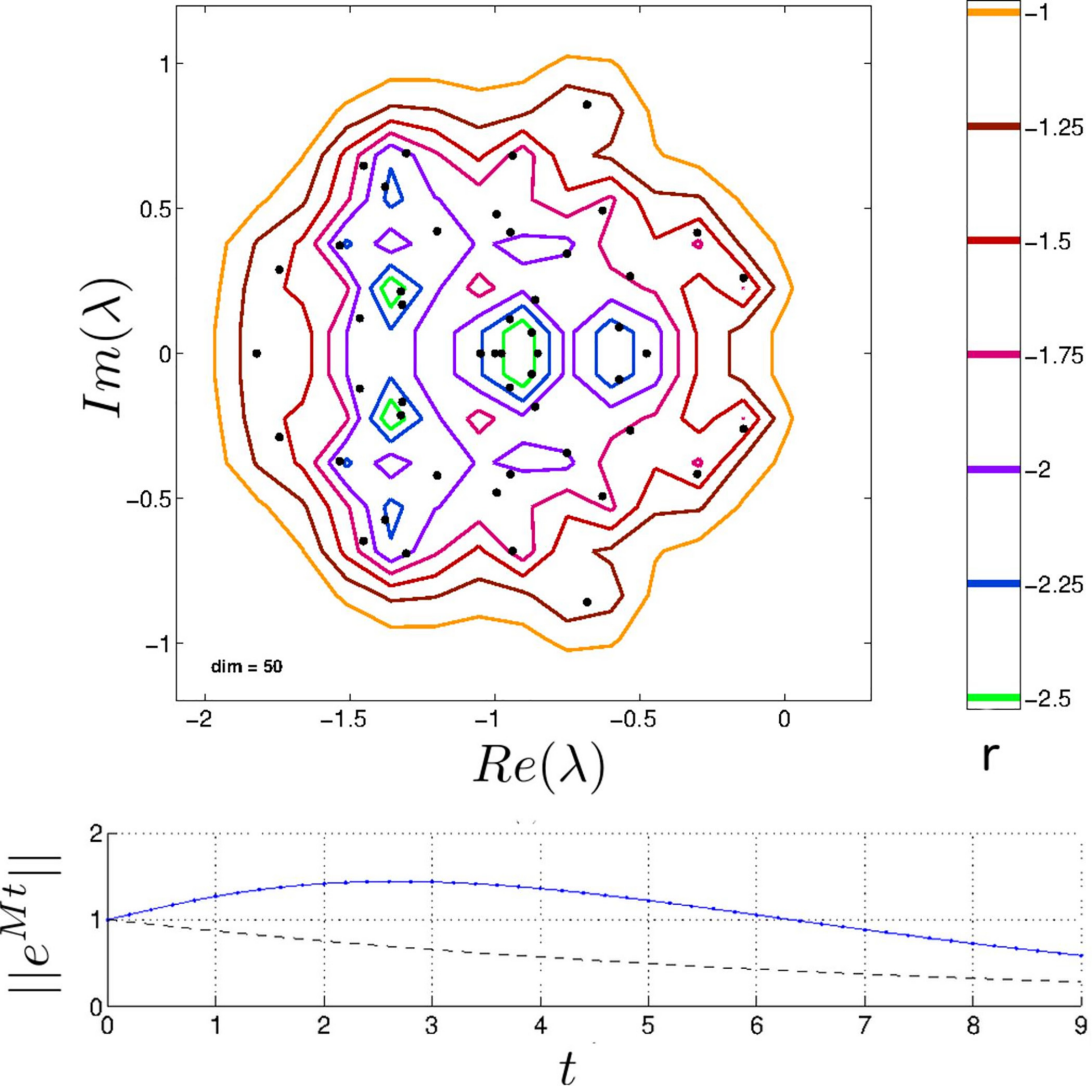
Top: Pseudospectrum of a random community matrix with S = 50, C = 0.1, μ = 1 and σ = 0.3, which is asymptotically stable. Contours in the complex plane illustrate the effect on eigenvalues of the community matrix M for noise of magnitude ϵ = 10r [31]. The contour for ϵ = 0.1 (i.e., r = −1) crosses the imaginary axis, implying that the pseudospectral abscissa is positive and so transient instability is observable. Bottom: Dynamics of ||eMt|| (arbitrary units of time, see Eq (9)). The dashed curve represents dynamics from eigenvalue analysis, whereas the solid curve represents dynamics predicted by positive ϵ-pseudospectral abscissa for ϵ ≈ 0.1.
